# Test–retest variability of microperimetry in geographic atrophy

**DOI:** 10.1186/s40942-020-00217-0

**Published:** 2020-04-30

**Authors:** A. Yasin Alibhai, Nihaal Mehta, Sheila Hickson-Curran, Carlos Moreira-Neto, Emily S. Levine, Elias Reichel, Jay S. Duker, Nadia K. Waheed

**Affiliations:** 1 New England Eye Center, Tufts Medical Center, Boston, MA USA; 2 Janssen Pharmaceutical Companies of Johnson & Johnson, Spring House, PA USA; 3 Department of Ophthalmology, Tufts Medical Center, 800 Washington Street, Box 450, Boston, MA 02111 USA

## Abstract

**Purpose:**

Microperimetry (MP) allows for measurement of retinal sensitivity at precise locations and is now commonly employed as a clinical trial endpoint. Test–retest reliability is important when evaluating treatment effects in patients with geographic atrophy (GA). This study aimed to determine the test–retest variability of MP in patients with moderate to severe GA using the MAIA MP device.

**Methods:**

In this prospective study, patients with a confirmed diagnosis of foveal-involving GA were enrolled. Participants performed three MP assessments of a selected eye over two visits with the Macular Integrity Assessment (MAIA) 2 instrument (Centervue, Padova, Italy) utilizing a wide 30° grid, consisting of 93 stimuli (Goldmann III) using a 4-2 representation strategy, encompassing the entire area of GA and beyond. Mean retinal sensitivity (MS) was expressed as an average threshold value (dB) for the entire field tested. Coefficients of Repeatability at a 95% level (CoR_95_) were calculated for Point Wise Sensitivity (PWS). Fixation stability (FS) was assessed by evaluating the area of an elliptical representation encompassing 95% of the cloud of fixation points (CFP) dataset generated by the MAIA MP, known as the bivariate contour ellipse area (BCEA).

**Results:**

A total of 8 subjects were enrolled (21 tests), with six subjects completing 3 MP assessments. BCVA in these patients ranged from 20/100 to 20/800. The mean area of GA was 18.7 ± 12.3 mm^2^. The average time to complete one MP assessment was 13 min 9 s and mean BCEA@95% was 38.5 ± 19.3°2. The MS was 14.3 ± 4.5 dB. No significant increase in MS was noted between testing pairs 1&2 and 2&3. The preferred retinal locus was maintained in the same quadrant on successive tests. The mean CoR95 for PWS were similar for testing pairs 1&2 (± 3.50 dB) and 2&3 (± 3.40).

**Conclusion:**

Microperimetry using a wide grid can be reliably performed in a reasonable amount of time in patients with moderate and severe vision loss secondary to GA. There was no learning effect seen between sequential assessments when analyzing MS or PWS. A change of approximately 4 dB in PWS provides a threshold for considering a true change in this patient cohort.

## Introduction

Microperimetry (MP) is a several-decades old technology designed to test retinal sensitivity at different points in the macula. First developed in the 1980s, MP was initially deployed as part of a scanning laser ophthalmoscope system. In its early forms, MP systems were relatively difficult to use. In the last two decades, however, there has been a resurgence in development of new microperimetry systems, beginning with the Nidek MP-1 and continuing more recently with the Nidek MP-3 and Macular Integrity Assessment (MAIA) 2 systems, which are more user friendly with the addition of features such as eye tracking.

With newer improvements, microperimetry (MP) has gained more widespread adoption as a means of functional retinal assessment. By presenting gradually decreasing intensities of light stimuli in a pointwise fashion, MP allows for precise interrogation of retinal sensitivity. When combined with fundus photography and other imaging modalities such as optical coherence tomography, MP can assist in the correlation of structural and functional deficits. Iftikhar et al., for example, recently applied MP in combination with SD-OCT and FAF to study the progression of retinitis pigmentosa [1].

Microperimetry is now also routinely employed in clinical trials as an endpoint; in the phase 3 clinical trial of lampalizumab, sensitivity assessed by microperimetry was used as a secondary endpoint in a subset of patients [2]. However, even at that time, the MP systems used lacked both the dynamic range as well as the ability to track the eye that the newer systems have. MP is particularly useful for the assessment of functional changes in disease states that manifest with local structural pathology, such as age-related macular degeneration (AMD). Changes in retinal function on MP have been shown to extend beyond the borders of GA, and changes in scotomatous points associated with GA growth, suggesting that MP may hold potential in better assessing the extent of visual loss and predicting progression in patients with geographic atrophy (GA) [3].

Given the utility of MP in the study of GA and evidence for variations in repeatability adjacent to scotomas, it is critical to better understand the repeatability of MP measurements in GA. Repeatability is an important metric in assessing the utility of MP for evaluation of treatment effects in patients with GA, and holds relevance in clinical trials using MP-derived sensitivity measurements as endpoints. This study looked to measure the test–retest repeatability of MP measurements in GA with poor visual acuity (20/80 or worse) using the MAIA MP device.

## Methods

### Inclusion criteria

This was a prospective study that enrolled subjects between the ages of 55 to 90, with a diagnosis of age-related macular degeneration with geographic atrophy (GA), and a best corrected visual acuity (BCVA) between 20/80 and 20/800 secondary primarily to the GA. Patients had no previous MP testing. Only one eye from each subject contributed to the study cohort. Subjects with age-related macular degeneration without geographic atrophy were excluded. Additionally, subjects with concomitant diagnoses as the primary cause of low vision were excluded, including diabetic retinopathy and anterior segment pathology.

### Laboratory tests

Refraction and High Illumination/Contrast ETDRS best corrected visual acuity (BCVA) was used to determine eligibility of each eye. Fundus autofluorescence (FAF) with the Heidelberg Spectralis was performed to document the area of atrophy after administering the MP testing.

### Testing protocol

Subjects underwent a short, uniform training session on the fellow eye prior to beginning testing, in which the use of the microperimeter was actively explained to them prior to and as they were taking the training module. Subjects underwent 3 MP assessments of the study eye over the course of two visits within a month of each other (test 1 and 2 on visit one and test 3 on visit two; or test 1 on visit one and test 2 and 3 on visit two). Examinations were performed by a trained operator (A.Y.A.) who encouraged the patients during the course of the testing and provided active instructions as necessary. Testing was performed using a custom built, foveal-centered wide 30º grid consisting of 93 white stimuli (Goldman III; 4 mm^2^, 25.6 arc minutes) presented for 100 ms each using a 4-2 ladder strategy. This grid encompassed the entire area of all GA lesions and beyond. Mean retinal sensitivity (MS) was calculated by the MP system and expressed as an average threshold value (dB) for the entire testing area. Fixation stability was assessed by evaluating the area of an ellipse encompassing 95% of fixation points (CFP), a metric generated by the MAIA MP in-built software and termed bivariate contour ellipse area (BCEA). Point Wise Sensitivity (PWS), a measure of retinal sensitivity at individual retinal locations, was independently calculated for all 93 stimulus points. Coefficients of Repeatability (CoRs), representing a value for which 95% of the test–retest differences for the same subject are expected to lie, was calculated for MS and PWS.

## Results

8 subjects were included in the study. A total of 21 MP assessments were performed, with 6 subjects completing all 3 exams and 2 subjects completing 2 exams. BCVA ranged from 20/100 to 20/800. The mean area of GA in this patient cohort was 18.7 ± 12.3 mm^2^. There was no correlation found between GA size and any MP performance metric or between baseline BCVA and MP performance.

The mean time to complete each MP assessment was 13 min and 9 s. Where subjects had completed all 3 exams, the average time for the first MP assessment was 13 min and 30 s, for the second MP assessment 12 min and 54 s, and for the third MP assessment 12 min and 48 s. There was no statistically significant difference observed between MP testing times.

Fixation stability (FS), assessed in our study by the mean BCEA@95% was 38.5 ± 19.3(º) [2]. FS was noted to worsen as the MP assessment progressed. For a complete set of three assessments, the average MS for the first assessment was 13.8 dB, for the second assessment was 13.9 dB, and for the third assessment was 14.98 dB. There was no statistically significant differences between the three sets of average MS results. Between assessment 1 and 2, the mean coefficient of repeatability at 95% (CoR_95_) for MS was ± 5.77. Between assessment 2 and 3, the CoR_95_ for MS was ± 8.91. And between assessment 1 and 3, the CoR_95_ for MS was ± 6.01.

The mean number of scotomatous points for the first set of MP assessments was 15, for the second set 18, and for the third set 16. The difference in mean scotomatous points between sequential tests was not found to be statistically significant.

For a complete set of three assessments, the average PWS for the first assessment was 15.2 dB, for the second assessment was 15.1 dB, and for the third assessment was 15.6 dB. There was no statistically significant differences between the three sets of average PWS results. Between assessment 1 and 2, the mean coefficient of repeatability at a 95% level (CoR_95_) for PWS was ± 3.50. The CoR_95_ were similar for testing pair 2 & 3 (± 3.40). Between assessment 1 and 3, mean CoR_95_ was ± 4.58 dB.

**Fig. 1:**
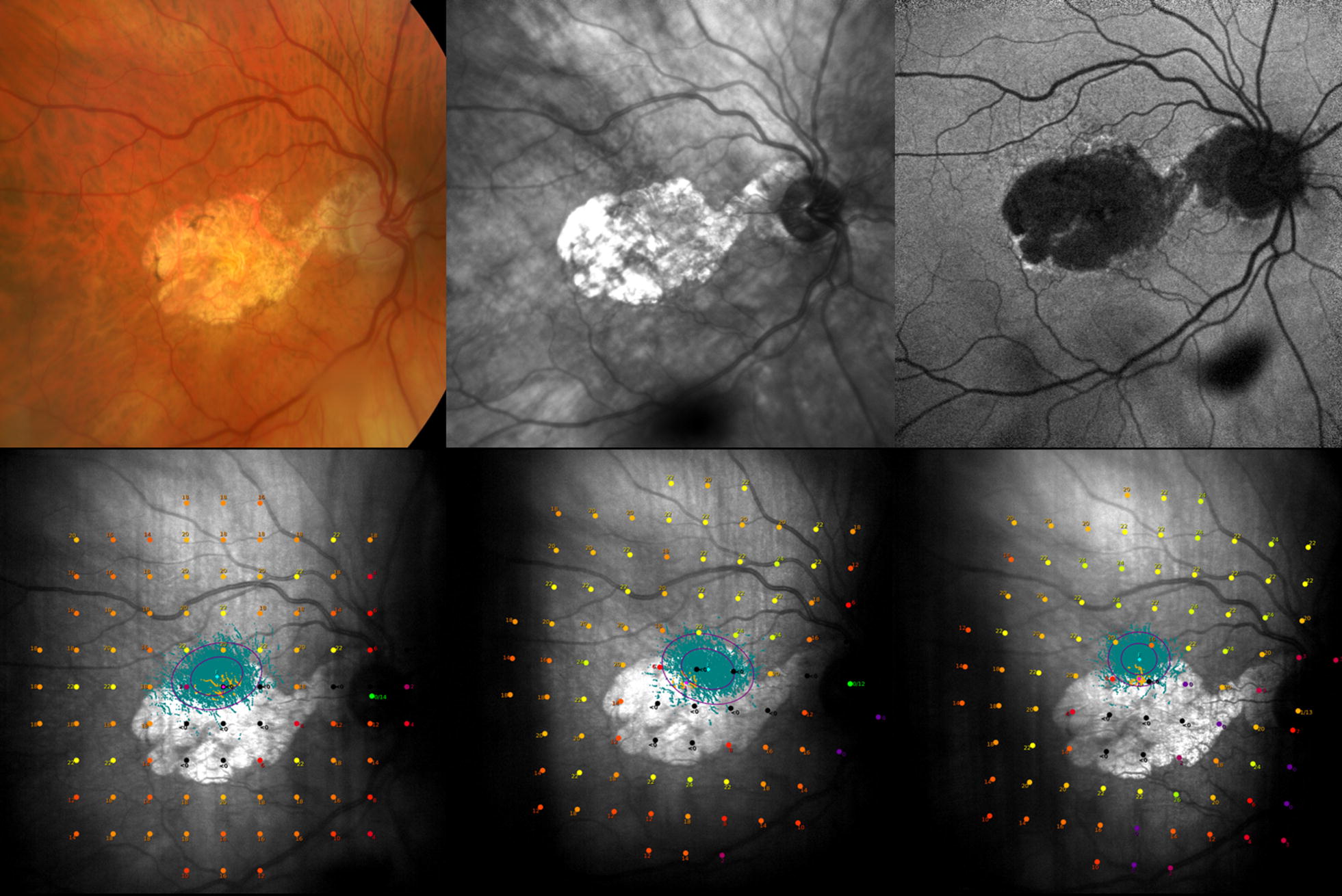
Top row: Multimodal imaging showing subject with GA; L-R: Color fundus, red free and auto fluorescence imaging. Bottom row: SLO images from MAIA device showing the 30° grid, consisting of 93 stimuli used during testing. Fixation Stability represented by green dots and purple ellipses encompassing a given proportion of the CFP dataset (BCEAs of 63% and 95%) L-R: Test 1–3 of one subject performed in follow-up mode

**Fig. 2:**
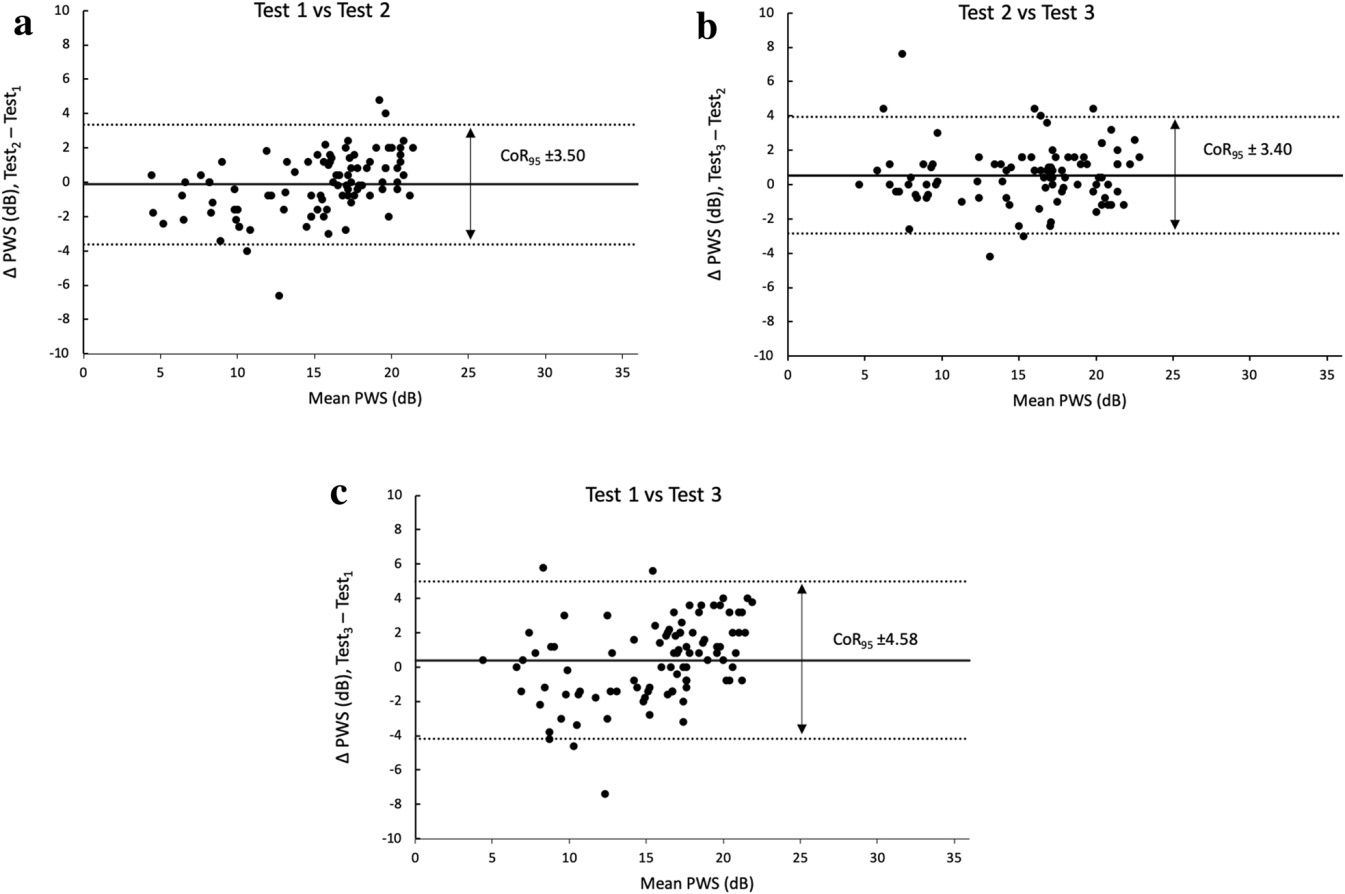
Bland–Altman plots of PWS, with horizontal dashed lines representing upper limits of 95% of the mean (+ 2 SD) and lower limits of 95% of the mean (− 2 SD) from top to bottom, respectively, and the horizontal solid black line representing the mean

As part of a sub-analysis assessing MS and PWS between MP performed on the same day (assessment 1 vs. assessment 2 when these were performed consecutively), the average MS for the first assessment 14.8 dB and 14.5 dB for the second assessment (no significant difference). The average PWS was 17.6 dB and 17.3 dB for the first and second assessments respectively, with a mean CoR_95_ of ± 3.58 dB (Figs. 1, 2).

## Discussion

This study is the first to investigate the variability of MP measurements performed by the same examiner over two to three testing sessions in patients with advanced Geographic Atrophy. These results indicate a high reproducibility in PWS values, of less than ± 4.0 dB, between successive tests, suggesting that a threshold change of ± 4 dB in PWS measurements of GA patients provides a reasonable benchmark for distinguishing true change from intrasession variability when studying how disease is advancing or the effect of interventions.

Prior studies examining the variability of MP measurements have generally shown a high degree of reproducibility in clustered or full-field measurements, such as mean sensitivity, but greater variability in PWS. Early studies of the MP-1 demonstrated relatively high repeatability and reproducibility in mean sensitivity (CoR_95_ < 2.0) in both normal and pathologic eyes, including those with intermediate AMD [4, 5]. Weingessel et al. did not report PWS but showed good repeatability in mean macular sensitivity, reporting standard deviations of 0.532, 0.830, and 0.589 for young normals, aged normals, and patients with AMD (intermediate AMD or foveal sparing GA), respectively, in intra-examiner variability. Chen et al. found a mean CoR_95_ for PWS of ± 4.96 dB in patients with a range of macular diseases. This is a significant variation given that the maximum PWS measurement in this study was around 20 dB. In a study by Cideciyan et al. using the MP-1 with retinal degeneration patients (ABCA4-associated retinal degeneration and retinitis pigmentosa), the CoR_95_ for PWS was ± 4.2 dB, similar to our findings. Studies done using the newer Nidek system, the MP-3, also showed that mean sensitivity repeatability was relatively high in normal and macular disease patients (GA, drusen maculopathy, or epiretinal membrane): ± 1.2 dB and ± 1.6 dB, respectively. However, as with the MP-1, PWS measurements remained variable with a CoR_95_ of ± 3.3 dB for healthy subjects and ± 5.0 dB in the macular disease cohort, placing the variability of PWS measurements in patients with macular disease in a range similar to studies on the MP-1 [6]. As Palkovits et al. point out, the MP-3 has a much wider dynamic range of stimuli than does the MP-1, minimizing the impact of a ceiling effect; moreover, this study used a more sensitive 4-2-1 staircase strategy, which would be expected to be more sensitive to variability in PWS measurements.

The MAIA system appears to have decreased variability in macular PWS as compared to the MP-3, with a CoR_95_ estimated at ± 3.81 dB in one study of normal eyes [7]. However, in the same study measurements taken at the border of the ONH, used as a model for peri-scotomal measurements, had a PWS CoR_95_ as high as ± 12.99 dB. Wu et al. used the MAIA to examine test–retest variability in MS and PWS in AMD patients without foveal GA and also found a relatively low CoR_95_ in normal and AMD eyes (± 2.01 and ± 2.32, respectively) [7]. A modified MAIA system was also applied by Welker et al. for mesopic and scotopic measurements in normal and intermediate AMD patients [8]. In the control and AMD groups, the CoR_95_ was ± 4.4 and ± 3.96, respectively, for mesopic measurements, and ± 4.52 and ± 4.56, respectively, for scoptopic. Taken together, these results from prior studies on the MAIA suggest that PWS can be measured using the MAIA system with a relatively high degree of repeatability even in diseased eyes, but that complete scotomas may increase the variability of peri-lesion measurements. Our results suggest that, at least in geographic atrophy patients, successive PWS measures can be obtained with a reasonably high degree of repeatability.

Notably, this study did not identify a learning effect between the testing pairs, which had similar CoR_95_ even for PWS. Although initial studies suggested a lack of a learning effect in MP [5, 9], the more recent study by Wu et al. using the MAIA found a significant increase in PWS between the first and second exam, indicating a possible learning effect despite a practice session before testing. The authors conclude that discarding the first exam may minimize intrasession variability. However, our results differ from the Wu et al. finding in that we did not see a difference in PWS between the different testing pairs. We think this could be explained by several reasons. One, perhaps the use of a guided training session on the fellow eye reduced variability. Additionally, there was active monitoring and guidance of the patients during their testing. This suggests the importance of good acquisition in reducing variability between scans. Lastly, a small learning effect could fail to reach significance with a limited sample size such as ours. Regardless, we do think good training and active monitoring can help reduce variability, and in the presence of conflicting data, it is probably best to discard the first test as a learning test when using MP in clinical trials and studies to assess sensitivity in GA.

In defining the level of analysis for microperimetry measurements, there is an apparent trade-off between variability and granularity. Mean sensitivity offers the most reproducible but least granular measurement, while PWS is the most granular but most variable level of analysis. Clustering measurements offers one way to improve repeatability while still allowing for distinctions to be made between areas of the measured region. Past microperimetry studies have used a foveal clustering with a 5º radius [1] and a para-foveal ring ranging from 2–4º to 3.5–4.7º [10, 11]. A high degree of repeatability in PWS, as was seen in this study, suggests less need for clustered measurements.

There were several limitations of this study. First, the sample size was relatively small (n = 8) and patients were examined on one device (the MAIA microperimeter). Future studies should further examine the repeatability of MP in other disease states on the MAIA, as well as on other devices for GA patients. In addition, this study was performed using one highly skilled operator, and did not compare between different operators.

## Conclusion

Microperimetry using a wide grid can be reliably performed in a reasonable amount of time in patients with moderate and severe vision loss secondary to GA. A change of approximately ± 4 dB in PWS provides a reasonable threshold for considering a true change in GA patients.
